# Fabrication of Polycrystalline Cubic Boron Nitride/Metal Composite Particles by Surface Metallization Followed by Electroless Deposition Technique

**DOI:** 10.3390/ma14247906

**Published:** 2021-12-20

**Authors:** Walid Mohamed Daoush, Turki Saad Alkhuraiji, Abdulrahman Dohymish Alshammri

**Affiliations:** 1 Department of Chemistry, College of Science, Imam Mohammad Ibn Saud Islamic University (IMSIU), Othman Ibn Affan Street, Al Riyadh 11623, Saudi Arabia; chemabdulrahmann@gmail.com; 2 Department of Production Technology, Faculty of Technology and Education, Helwan University, Saray–El Qoupa, El Sawah Street, Cairo 11281, Egypt; 3 National Center for Irradiation Technology, King Abdulaziz City for Science and Technology, Al Riyadh 11442, Saudi Arabia; khuraiji@kacst.edu.sa

**Keywords:** cubic boron nitride, composites, abrasives, molten salt reaction, Ti metallization, Tin/Ag metallization, electroless powder coating

## Abstract

Polycrystalline cBN/copper composite abrasive particles were prepared by an electroless powder coating process. Ti metallization and tin/silver metallization techniques were used to improve the coating process by depositing an autocatalytic metallic layer on the surface of the cBN particles. Metallized, as well as un-metallized, cBN particles were further coated by copper using electroless deposition. Electroless copper coating of un-metallized and metallized cBN particles by 90 wt.% of copper were achieved. The surface morphology, the composition and the crystalline phase identifications of the metallized cBN particles, as well as the 10 wt.% cBN /copper composite powders, were investigated by field emission scanning electron microscopy, an energy-dispersive spectrometer and an X-ray diffractometer. The results show that the surface of the Ti metalized and tin/Ag-metallized cBN particles were covered by the nanosized Ti or Ag layer, respectively, which enhanced the deposition of the copper during the electroless deposition bath. The results also showed that the deposited layer on the metallized cBN particles was composed mainly of metallic copper. The produced 10 wt.% cBN/copper composite particles also underwent thermo-gravimetric analysis to investigate its stability at high temperature. It was revealed that the Ti-metallized cBN/copper composite powder has higher stability at 800 °C under the environmental conditions than the tin/silver-metallized and the un-metallized cBN/copper composite particles, respectively.

## 1. Introduction

Abrasive materials have several important characteristics, such as high hardness and longevity; quick cutting or wear of surfaces and stiffness are also important for applying specific forming or cutting techniques, especially in the production of pipes and wires. The tribological surface properties of the abrasive particles are especially crucial for subsequent uses to control the wear and tear processes. Thus, the hardness of the materials determines the installations and the function of technical systems [1].

Different minerals in nature are used as abrasive materials. Silica, alumina and silicon carbide are the most common minerals used in the production of cutting tools, sandpapers, grinding wheels and other abrasive products [2]. Different synthetic materials are used as abrasive particles [3]. The synthetic abrasives and their application can be categorized in three groups. The first group is the silicon dioxide (SiO_2_) and silicon carbide (SiC) group, which is mainly used in the fabrication of cutting wheels and papers. The second one is the fused aluminum oxide (Al_2_O_3_) group, which is composed mainly of calcined bauxite raw material at ~950 °C after removing the free capillary and the chemically combined water of hydration. The third group is the super hard materials, such as diamond and cubic boron nitride (cBN) [3]. 

Recently, cBN has been used as an alternative material to diamond for the cutting of ultra-hard materials. This type of high-performance cutting tool material has the highest hardness at moderate and high temperatures due to the combination of cBN with suitable binder; however, it causes little chemical interaction between the material compose and the working piece [4]. cBN has superior thermal conductivity, high chemical stability, and great rigidity at moderate and high temperature. It is commonly used as a suitable material for cutting hard steel, cast steel, iron or other thermally stable materials, because they have high stability when they interact with ferrous materials at high temperature and high cutting speed. On the other hand, it has become the most promising tool after diamonds, which are fully compensated. A disadvantage of diamonds is that they are not suitable for cutting iron base and steel materials, because the bonding in the cBN is a high-strength covalent bonding between carbon and nitrogen atoms. It is difficult to sinter a single crystal of cBN, because it requires controlling the sintering conditions at temperatures above 2000 °C, under pressure above 7 GPA, and several problems are likely to occur during the fabrication process of the material. Therefore, the method of synthesis of polycrystalline boron nitride/metal matrix composite material was suggested by researchers to sinter cBN particles combined with a suitable amount of metallic binder in order to fabricate the cBN/metal matrix composite and avoid the sintering conditions at high temperature and high pressure (HTHP) [5]. It was reported that the metal binder plays an important role in the consolidation of cBN at high temperature and high pressure. Addition of an appropriate amount of metallic binder can not only reduce sintering temperature and pressure, but also improve the physical and mechanical properties, like stiffness, of the sintered bodies of the composite materials [5]. This is achieved by high pressure sintering, hot isostatic pressing, spark plasma sintering and infiltration. Spark plasma sintering at 1423 K can be used to fabricate copper/diamond composites with thermal conductivity of 690 W/m K. In the previous work of Yoshida and Morigami, diamond/copper composite materials were produced by high pressure sintering at 4.5 GPa [6]. However, the methods mentioned above cannot avoid overheating under some severe conditions, including high sintering temperature above 1000 K under high pressure for limited application to obtain diamond/copper composites with precise thermal properties [7]. Several kinds of light metals, such as Al, and also refractory metals, such as Ti, W, and Hf, are often used as a metallic binder for improving the sintering process of cBN products. Aluminum, because of its low melting point, is widely used as the best binder among all the metals. Under high temperature, aluminum is melted and forms a liquid metallic binder phase to fill the gap between cBN reinforced particle and form the ceramic phase of AlN and AlB_2_, which prevents the phase transition from the cubic phase (cBN) to the hexagonal phase (hBN). In the preparation process of the cBN/Ti composite materials, Ti is very reactive and easily reacts with cBN to form TiN and TiB_2_. The products have high hardness and high chemical stability at high temperature, which can enhance the thermal stability, red hardness and breakage cBN hardness. Tungsten metal can be used as a metallic binder of the cBN due to its high melting point of 3410 °C, its compatibility with other elements, and the fact that its thermal expansion coefficient is matched with the cBN. Addition of tungsten can greatly reduce the modulus of expansion of the metallic binder, reduce heat stress and reduce incidence of cracks in the bulk material [8,9,10,11].

Powder coating is a good technique that was developed to load some metals on the surface of the ceramic particles and to improve the poor wettability and weak binding between ceramic particles and the metal matrix of the binder phase of the composite. One method of powder coating is the electroplating method. Electrolytic powder coating is a process of using a metal or alloy matrix containing solid particles such as Al_2_O_3_, TiO_2_, SiC, WC, or SiO_2_ as a reinforced phase. Several decades of great research have been focused on the influence of electrical parameters, such as the electrolysis conditions, type of the electrolyte, additives, temperature, pH, the current density, reinforcement properties, type, size, shape, conductivity, surface charge, morphology and properties of coatings [12,13]. In addition, the decrease in the size of the particles used as a reinforcement phase increases the tendency of agglomeration of particles, due to the enhancement of the surface energy, while reducing the content in the metal and intermediate arrays of the size of the grains of the matrix [14]. Previous research has reported that the microstructure and properties of metal deposits may change under the application of particular current conditions. Other research work concerns the coating of reinforced particles by Ni-P composite coatings; most of this research is concerned with electroplating to obtain its deposits with enhanced features [15]. The effects of electrolysis parameters on the structure and morphology of Ni coating of Ni-P matrix complex reinforced with TiO_2_, SiC and WC particles were also examined in several previous reports [16]. Various coated layered materials developed by mechanical milling, including boron nitride, graphite and molybdenum disulfide, have been successfully exfoliated by a ball milling method with polycation solution as the medium, which can effectively stabilize the layered materials in the solution [17,18]. Other researchers have developed methods used to coat ceramic particles with metals and its alloys by chemical reduction of the metallic ions in solutions, e.g., the electroless deposition technique, which is a simple and novel fabrication process of coating different kinds of reinforced particles, fibers and whiskers. The process is based on using the electroless metal deposition technique to encapsulate reinforcement particles by a metallic nano layer at room conditions. The development of such composite coated powders can be easily incorporated with a variety of metals, such as Cu, Ti, Ag, Ni, Pd, Pt and its alloys [19,20,21,22,23,24,25,26,27,28,29]. 

In the present work, the authors aimed to study the effect of the surface modification by the metallizing process of the surface of cBN abrasive ceramic particles with different methods, namely Ti metallization by molten salt process and tin/silver metallization followed by coating of the surface metallized cBN particles by the electroless copper deposition technique. The morphology, as well as the compositional analysis, of the produced cBN/Cu composite powders were investigated by using the field emission scanning electron microscopes supported by energy dispersion X-ray spectroscopy. The crystalline phase identification was also investigated by an X-ray diffraction instrument and the crystallite size was estimated using the X-ray line broadening method by applying the Scherrer’s equation. The investigated particles underwent thermal analysis to study their stability at high temperature. It was expected that introducing a thin metallic layer on the surface of the cBN particles would improve the sintering process of cBN at normal conditions of moderate sintering temperature and compaction pressure, which would allow one to avoid using high temperature and high pressure techniques. In addition, it was expected to improve the physical and mechanical properties of the sintered bodies of the cBN/metal matrix composite materials by the conventional powder metallurgy process.

## 2. Materials and Methods

### 2.1. Materials

Analytically pure chemicals and polycrystalline cBN abrasive particles were used in the experimental work. Polycrystalline cubic boron nitride powder of particle size ~220 μm was purchased from Iljin Diamond Co., Ltd., Seoul, Korea. KCl, NaCl and K_2_TiF_6_ were purchased from Aldrich Co., Ltd. Darmstadt, Germany. Silver nitrate and tin chloride were provided from BDH Chemicals Ltd., Poole, UK. Formaldehyde and sodium hydroxide was supplied by Panreac E.U. Castellar del Vallès, Spain. Copper sulphate pentahydrate was purchased from Winlab Co., Ltd., Market Harborough, UK. Sulphuric acid, hydrochloric acid and ammonia solution were provided from Riedel de Haen, Seelze, Germany. Potassium sodium tartrate was purchased from Meric Co., Holzgerlingen, Germany. 

### 2.2. Methods

The experimental work aims to produce 10 wt.% (cBN)/copper abrasive composite particles by using two different surface metallization techniques, namely Ti metallization, tin sensitization/silver metallization, followed by powder coating of the metallized and un-metallized cBN particles with an electroless copper deposition process.

#### 2.2.1. Powder Cleaning by Chemical Treatment 

The cBN abrasive particles underwent acid-treatment by using 33 vol.% concentrated hydrochloric acid as an aggressive chemical at room temperature to remove any foreign inorganic impurities from the cBN powders. The obtained powder was washed by distilled water to remove any remaining acid. This chemical treatment process helps to improve the properties of the surface of the cBN abrasive particles and may change their surface chemistry to enhance the coating process and improve the adhesion between the cBN particles and the metallic coating layer.

#### 2.2.2. Ti Metallization by Molten Salt Process

**Figure 1:**
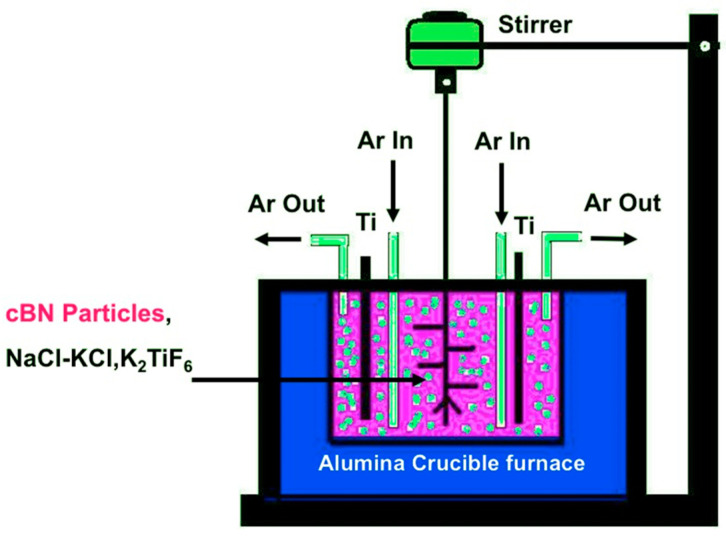
Experimental setup of the Ti-metallization process of the cBN particles by molten salt reaction at 900 °C.

The obtained acid-treated cBN abrasive particles underwent surface metallization by using a molten salt Ti deposition process. The investigated cBN particles were mixed separately with an equal amount of the molten salt reaction mixture, which was composed of 40 wt.% KCl, 40 wt.% NaCl, and 20 wt.% K_2_TiF_6_ in a suspended medium of 100 mL highly pure ethanol. The mass ratio of the cBN particles to the Ti metal was adjusted in the molten salt reaction process to be 1:10. The obtained powder of the reaction mixture was dried at 80 °C for 30 min to evaporate the ethanol. The dried reaction mixture was blended with an appropriate amount of sponge Ti metal powder, spread inside alumina crucible and heated in a closed furnace as shown in Figure 1 for 1 h under argon atmosphere with a flow rate of 0.5 L/min at 900 °C and a heating rate of 10 °C/min. After the reaction was completed, the sample was cooled within 8 h inside the furnace. Then the sample was removed from the furnace and subjected to ultrasonic cleaning at 40 kH in a sufficient amount of distilled water at 50 °C for 3 h in order to separate the reaction products from the remaining spongy Ti particles. The produced powders underwent washing with double distilled water, filtration and acid treatment with 10 vol. % HCl to dissolve any remaining salts from the side reactions of the molten salt process. The produced powder was subsequently washed again by double distilled water for several times and dried under vacuum at 80 °C for 2 h. 

#### 2.2.3. Silver Metallization by Tin/Silver Method

About 1.17 g of SnCl_2_·2H_2_O powder was dissolved in 10 mL of water, and HCl was used to adjust the pH at 1.5. The obtained acid treated cBN particles (0.1011 g) was added to the solution and stirred by a magnetic stirrer for 15 min. The suspended cBN particles were collected from water by decantation, and the collected cBN particles were washed several times with double distilled water. AgNO_3_ (0.227 g) powder was dissolved in 100 mL of double distilled water, Liquid ammonia was used to adjust the pH of the solution at 11.4. The obtained solution was stirred by a magnetic stirrer for 15 min before 10 mL of 38 vol.% formaldehyde solution was added dropwise until the color of the solution changed to grey. Then the remaining solution was filtered and the obtained coated powder was dried at 130 °C for 30 min. The dried powder was collected and stored.

#### 2.2.4. Electroless Cu Coating of cBN Abrasive Composite Particles

**Figure 2:**
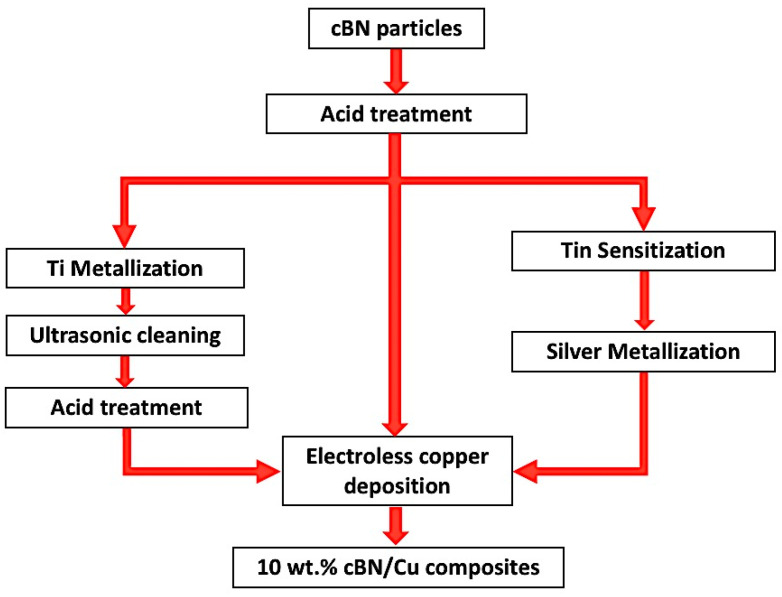
Schematic flowchart of the polycrystalline cBN/Cu composite particles fabrication process.

The acid-treated, Ti-metallized, and silver-metallized cBN particles underwent surface coating by electroless copper chemical deposition to prepare 10 wt.% cBN /copper abrasive composite particles. In a 250 mL beaker, 0.35 g of CuSO_4_·5H_2_O was dissolved in 10 mL of double distilled water and the solution underwent stirring using magnetic stirrer at 500 RPM under normal conditions of pressure and temperature. Potassium sodium tartrate (1.7 g) was added to form a complex with the copper ion and prevent precipitation of copper as copper hydroxide in the alkaline media. The pH of the solution was adjusted at ~12.5 by adding a required amount of NaOH. A calculated amount of cBN of 0.01 g equivalent to 98 wt.% was added in the solution of the reaction, which was then continuously magnetically stirred for 15 min until the mixture was conducted in order to disperse the cBN particles in the solution of the reaction. About 10 mL of 38 vol.% formaldehyde solution was added to deposit the copper metal by reducing the dissolved copper ions in the solution on the surfaces of the cBN particles. The electroless copper deposition reaction on the surface of cBN particles was started and completed within 10 min from the addition of the formaldehyde. The solution was filtered and the produced copper coated cBN particles were dried at 110 °C. The above-mentioned procedure was conducted similarly for coating the tin/silver activated cBN particles. On the other hand, a copper coated sample containing un-metallized cBN abrasive particles was prepared as a comparative reference sample of the process. Figure 2 shows a complete schematic flowchart of the experimental setup of the fabrication process of the 10 wt.% cBN/copper abrasive composite particles.

#### 2.2.5. Powder Characterization

The morphology, particle shape, size and compositional analysis of the deposited metallic layers on the cBN particles surfaces were investigated by field emission scanning electron microscopy (FESEM) using model JEOL JSM-7600F and JEOL JSM-IT300LV, Japan, combined with energy-dispersive X-ray spectroscopy (EDS) using model Aztec Energy IE250, Oxford Instruments, UK. 

The crystal structure and phase identification of the investigated, as well as the metallized, cBN particles and the 10 wt.% cBN/copper coated composite powdered samples with different treatment and metallization processes were investigated by X-ray diffractometer using model Bruker D8 discover. The uncoated, as well as the coated, cBN particles underwent thermo-gravimetric analysis using a TGA instrument model Pyris 1 TGA, Perkin Elmer. The heating conditions were optimized at 10.00 °C/min heating rate in a temperature range between 25 °C and 800 °C under a highly pure nitrogen gas (99.999%) as a heating atmosphere. 

## 3. Results and Discussion

The coating process of nonconductive ceramic particles, such as the investigated cBN abrasive particles, is highly dependent on the pretreatment conditions of the surface of the particles. Although the composition of the coating bath of any coating process is the most visible and complex for any process, the problem is not due to the chemistry of the bath itself, but is more likely due to the pretreatment of the system used. In this investigation, several pretreatment and metallization processes were carried out on the surfaces of the cBN composite abrasive particles in order to improve the coating process.

### 3.1. Chemical Treatment of the cBN Abrasive Particles

**Figure 3:**
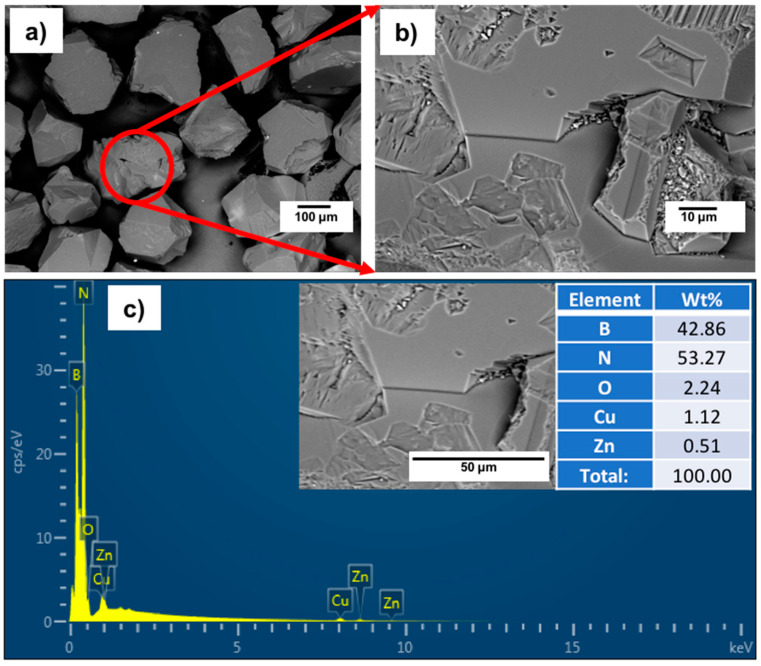
(**a**,**b**) FESEM images with different magnification and (**c**) EDAX compositional analysis of the investigated polycrystalline cBN abrasive particles.

Figure 3a,b shows typical FESEM images with different magnifications for the investigated polycrystalline cubic boron nitride (cBN). It was observed from the results that the investigated cBN abrasive particles have mean particle sizes of ~220 µm of irregular particle shape. Figure 3c shows the EDAX compositional analysis of the surface of the cBN particle. It was further observed that two main types of peaks were detected, which indicated that the particle is composed mainly of elemental boron and nitrogen. The first pretreatment step of the cBN abrasive particles before the metallization and coating processes occurred by employing aggressive chemicals to clean off and remove any foreign inorganic matter that contaminated the cBN particles [10]. Although cBN particles are chemically stable against usual acids, concentrated hydrochloric acid was used in this investigation to clean its surfaces from any foreign inorganic impurities. It was reported in previous work that boron nitride is a refractory material with high chemical resistance. It does not interact with acids like HCl, HNO_3_ and H_2_SO_4_, but it can react with molten liquids of LiOH, KOH and NaOH [11,12,13]. 

**Figure 4:**
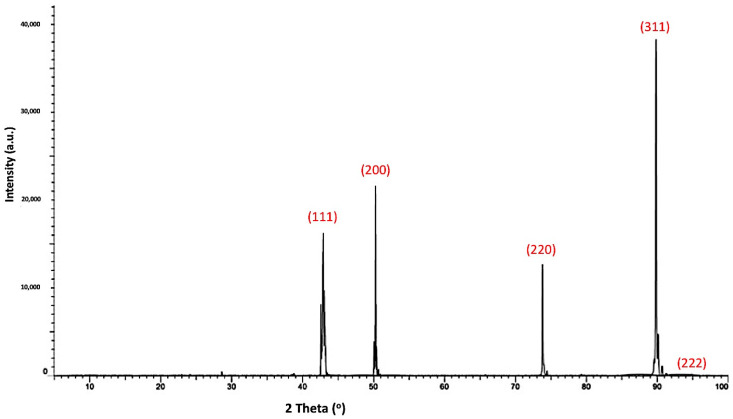
X-ray diffraction pattern of the as received uncoated polycrystalline cBN particles corresponding to JCPDS card number 25-1033.

Figure 4 shows the X-ray diffraction pattern of the as-received polycrystalline cBN abrasive particles. It was revealed from the results that the (111), (200), (220), (311) and (222) peaks corresponding to the JCPDS card number 25-1033 appeared due to the presence of the cubic phase of the polycrystalline cBN [22]. The crystallite size of the cBN abrasive particles was calculated at the highest intensity main peak of 2 *θ* = 90° by the X-ray line broadening method using Scherrer’s formula (*D* = 0.9λ/*B* cos *θ*), where *D* is the crystallite size, λ is the wavelength of the radiation, *θ* is the Bragg’s angle, and *B* is the full width at half maximum of the peak. The Scherrer crystallite size of the investigated cBN abrasive particles was estimated at 7.6 × 10^2^ Å at the main peak of the X-ray diffraction pattern [21].

### 3.2. Surface Metallization of cBN Abrasive Particles 

#### 3.2.1. Ti Metallization 

The surface of the treated polycrystalline cBN abrasive particles was metallized by depositing a conductive metallic Ti nanolayer. The Ti molten salt process was used to introduce an autocatalytic promoter layer to enhance the electroless copper deposition of the nanosized copper particles on the nonconductive ceramic surfaces of the cBN abrasive particles. In the molten salt reaction process, Ti metal was deposited on the surface of the cBN abrasive particles by heating the constituent of the molten salts chemical reaction under an inert atmosphere of Argon at 900 °C for 1 h. 

**Figure 5:**
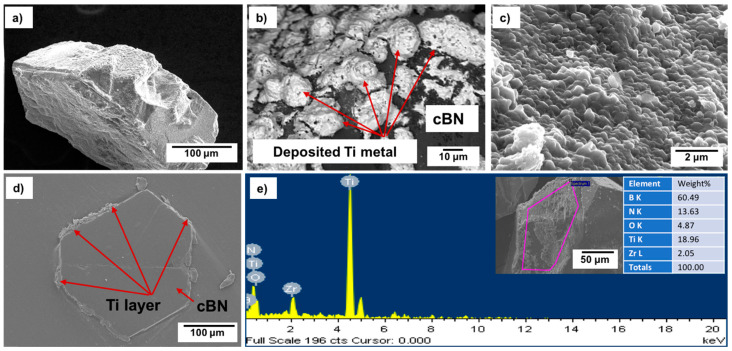
(**a**–**c**) FESEM images with different magnification, (**d**) cross sectional area and (**e**) EDAX compositional analysis of the Ti-metallized cBN particles.

Figure 5a−d shows FESEM images with different magnifications of the surface morphologies, particle size and shape, as well as the cross-sectional area of the deposited Ti layer on the cBN abrasive particles after surface cleaning with sonication using HCl solution. From the preliminary studies, it was determined that the remaining molten salt complex layer that adhered to the cBN particles could be dissolved in 10 vol.% HCl solution within 30 s under the effect of the ultrasonic waves at a frequency 40 kHz. Cleaned composite particles composed of Ti-metallized cBN abrasive particles were obtained. It was further observed that metallic Ti deposited on the surface of the cBN abrasive particles via the molten salt method; the cBN particles surfaces were successfully covered with a very fine and dense nano-sized metallic Ti layer. Although, as shown from the surface morphology, some agglomerated Ti particles of particle size in the microscale were deposited on the surface of the cBN particles (see Figure 5b), the deposited Ti metal is mainly in the form of nanosized Ti nanoparticles covering the surfaces of the cBN abrasive particles as a primary layer (see Figure 5c). The EDAX compositional analysis of the deposited layer, shown in Figure 5e, indicated that the uniform layer was composed mainly of metallic Ti that metalized the surface of the cBN abrasive particles. Two main peaks were observed, the first one for the elemental nitrogen and the second for the elemental titanium. Low intense peaks also appeared, indicating the presence of some trace element, such as zirconium, contaminating the metallic Ti coating layer due to the side chemical reaction that takes place between the molten salts reactants and the crucible of the heating furnace [22].

**Figure 6:**
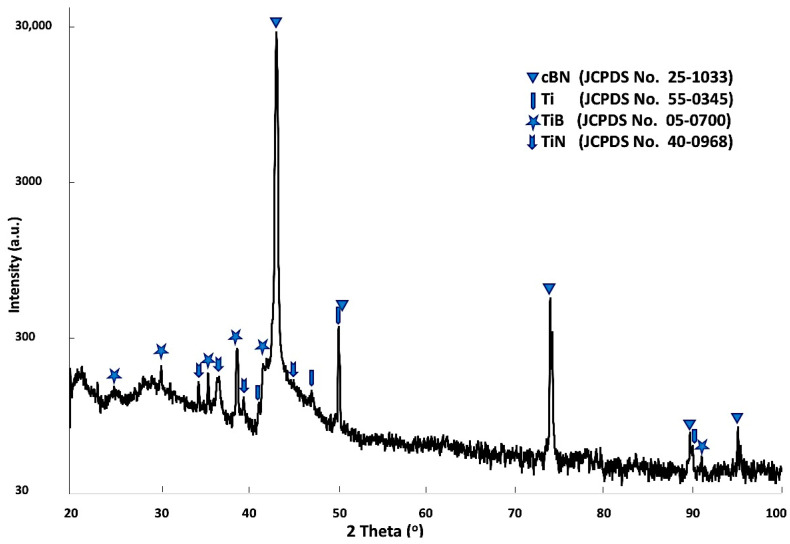
X-ray diffraction patterns of Ti-metallized cBN abrasive particles by molten salt reaction.

Figure 6 shows X-ray diffraction patterns of the Ti-metallized cBN particles by the molten salt reaction. It was observed from the results that four types of peaks were detected. The first type of peak, which is the main type of peak, was due to the presence of the cBN. The intensity of the (311) peak at 2θ = 90 ° was significantly decreased compared with the corresponding peak of the uncoated cBN particles (see Figure 4). This may be due to the effect of the deposited Ti layer on the surface of the cBN, which can decrease the diffraction intensity by changing the orientation of the incident X-ray beam. In addition, broadening of the peaks could also be due to the micro-strain enhanced in the cBN particles by heating during the reaction temperature of the molten salt process. The second type of peak corresponds to the JCPDS card number 55-0345 and appeared due to the formation of the deposited Ti metallic layer on the surface of the cBN abrasive particles. The third type of peak corresponds to the JCPDS card number 05-0700 of the TiB phase. The forth peaks corresponds to the JCPDS card number 40-0968 of the Ti_3_N_2-x_ phase. It was revealed from the results that an interaction took place between the surface of the cBN particles and the deposited Ti-layer under the molten salt reaction conditions. The formation of the two new compounds of TiB and Ti_3_N_2-x_ provide good evidence for the formation of an interface layer between the deposited titanium layer and the surface of the cBN particles. This interface layer is responsible for the binding and the enhancement of the adhesion between the surface of the cBN particles and the deposited layer of titanium [22]. 

In the Ti metallization process of the cBN particles by molten salt reactions process at a high reaction temperature of 900 °C, K_2_TiF_6_ dissolved into the reaction mixture of NaCl/KCl molten salts and the Ti^4+^ cation was produced. K_2_TiF_6_ was added to the molten salt mixture to enhance the Ti atoms and reacted with Ti^4+^ cations from the decomposed K_2_TiF_6_ and produce Ti^4+^. Then, the interaction of K_2_TiF_6_ with the NaCl/KCl molten salt mixture helped in the formation of titanium cations. The coating process began with the deposition of Ti^4+^ from the molten salt onto the surface of the cBN abrasive particles. This was followed by a disproportionation reaction on the surface of the cBN abrasive particles, whereby Ti^2+^ was both reduced to produce Ti and oxidized to produce Ti^4+^. It was supposed that the Ti deposition occurs in the molten salt bath is a polystep process and the slowest step is the rate determining step. The deposition rate of Ti metal on the cBN particle surface at certain temperature indicates that the deposition process may not be controlled by the diffusion process. It is reasonable to suppose that the disproportionation reaction of titanium may be the rate-determining step of the whole process; thus, sponge metallic titanium is required to add in the molten reaction in order to produce Ti^3+^ cation, which is needed for enhancing and continuing the molten salt reaction [22].

#### 3.2.2. Tin/Silver Metallization

This process is used to impart a uniform thin film composed of deposited metallic silver nanoparticles on the nonconductive surface of the cBN particles (energy band gab ~6.4 eV) [30], which ensures uniform adsorption of the subsequent coating and which therefore promotes a better coating process. The need to achieve selectivity in the coating process favors the use of the auto-catalyst promoters. In this investigated step, an auto-catalytic promoter was employed to the cBN abrasive particles, namely silver. The deposited silver particles improve the surface conductivity of the cBN particles (electrical resistivity > 10^15^ Ω·cm [30]) and enhance the electroless deposition process of copper on the surface of the cBN particles [10,11,12,13].

The tin sensitization method is used as a pretreatment step of the cBN particle surfaces to initiate the reduction of the silver ions to silver atoms that can be deposited on the nonconductive surface of the cBN particles. The sequential tin, then silver (so-called “two-steps”) catalyst system was investigated and was proven reliable and effective in metallization of nonconductive ceramic surfaces such as the surface of the cBN particles [23,24,30]. The tin ions adsorbed on the cBN particles surfaces reduced the silver ions in the 2nd step according to the following equation:

Sn^2+^ + 2Ag^+^ → Sn^4+^ + 2Ag^0^ (1)

The chemical reduction reaction of the silver nitrate in alkaline ammonia solution [Ag (NH_3_)^2+^/Ag + 0.373 V] into silver metal by using formaldehyde as a reducing agent successfully occurred on the surface of the tin sensitized cBN particles. As can be seen, the deposition of metal nanoparticles is achieved via the redox reaction, in which the cBN act as a cathode for the silver metal deposition from the reduction of the Ag^+^ metal ions in solution. Therefore, the process should allow electroless deposition of silver metal on the sensitized cBN particles surfaces [24]. The produced metallic silver atoms that were deposited on the surface of the cBN particles covered the surface by a very thin layer of conductive silver layer, improving the surface properties of the cBN particles and promoting the autocatalytic deposition reaction of the nanosized copper particles of the next step. 

#### 3.2.3. Electroless Copper Coating of Polycrystalline cBN Abrasive Particles

The un-metallized, Ti-metallized and tin/silver-metallized polycrystalline cBN particles underwent an autocatalytic coating by the electroless deposition of copper on its surface by the autocatalytic effect in the alkaline tartrate copper sulphate bath. The Ti metallization and the tin/silver metallization processes improve the surface chemistry and morphology of the cBN particles. It was expected to improve the coating process of the cBN abrasive particles by imparting a conductive Ti or Ag layer on the surface of the cBN particles, which ensures the subsequent deposition of a uniform copper layer. 

Chemical deposition of the coating layer composed of fine copper particles in the nanoscale has been widely studied in relation to the solution composition and process kinetics [18]. The minimum components of a solution are a soluble salt as a source of the ions of the deposited metal and a reducing agent used to reduce the ions to the metallic atoms. The rate of copper deposition can be affected by the reaction conditions, such as copper ion concentration, temperature, hydrogen ion concentration, and the type of complexing agent used to prevent the copper hydroxide deposition in the alkaline media. The source of copper could be a simple copper salt, such as copper sulfate, chloride, or nitrate. Various common reducing agents have also been suggested for use in the baths of the chemical precipitation, namely formaldehyde, hypophosphite, hydrazine, sugar, and dithionite compounds. When formaldehyde solution is utilized as a reducing agent, the pH of the solution should be adjusted above 12~13 at room temperature and pressure. Because simple copper salts are insoluble at pH above 4, the use of a complexing or chelating agent becomes necessary. The complexing agent could be one of the following groups of compounds: Tartrate salts, Alkanol amines, or EDTA (ethylendiamine, tetra-acetic acid) [19,20].

In the present investigations, a mixture of two chemical solutions A and B was used. The first solution was made by dissolving the equivalent amount of the CuSO_4_·5H_2_O with the tartrate chelating agent in aqueous media composed of potassium sodium tartrates alkaline solution and the pH was obtained by adjusting at 12.5 by NaOH solution. The second solution used formaldehyde as a reducing agent of the copper ions from the copper sulphate solution to copper metal. As five parts of solution A were added to one part of solution B at ambient temperature, the formaldehyde solution was dissociated in the alkaline media, forming nascent hydrogen atoms, which are responsible for the reduction of the copper ions to metallic copper atoms; the reaction started and completed within 10~15 min. The precipitation reaction of copper on the surface of cBN abrasive particles was almost completed, and the solution color changed from blue to colorless. The precipitation reaction depends mainly on the pH of the solution and the amount of formaldehyde. Once the pH of the solution reaches 12.5, copper precipitates on the surface of the cBN particles in the solution. However, in case of the Ti and tin/silver-metallized cBN abrasive particles, the reaction rate occurred faster than the reaction on the surface of un-metallized cBN abrasive particles. This is due to the fact that both the titanium as well as the silver layer enhanced the autocatalytic reaction of the electroless copper deposition on the Ti-metallized and silver-metallized cBN particles, respectively.

The Cu-precipitation is believed to proceed according to the following reaction:

Cu^2+^ + 2HCHO + 4OH^−^ → Cu^0^ + H_2_ + 2H_2_O + 2HCO_2_^−^ (2)

By changing the pH of the bath, it was found that the rate of Cu-precipitation was increased by increasing the pH to a maximum at ~12.5 and then decreased further on. It was reported that the Cu-coating chemical process of a similar composition that was used in this investigation is considered as an autocatalytic process [20,21]. However, it was found that Cu-coating is deposited on any surface found in the solution once its pH value becomes above 12.5. It is believed that the Cu-coating process used in this investigation is a chemical reduction process for Cu-ions by formaldehyde, which served as a reducing agent. As a result, both Ti-metallized and tin/silver-metallized cBN abrasive particles were coated faster than the un-metallized cBN abrasive particles [18,20]. The deposited titanium or silver nanoparticles on the surface of the cBN abrasive particles convert the nonconductive surface to a conductive one, which can act as a promotor to enhance the autocatalytic effect of the deposition of copper nanoparticles on the surface of the cBN abrasive particles [12,30].

**Figure 7:**
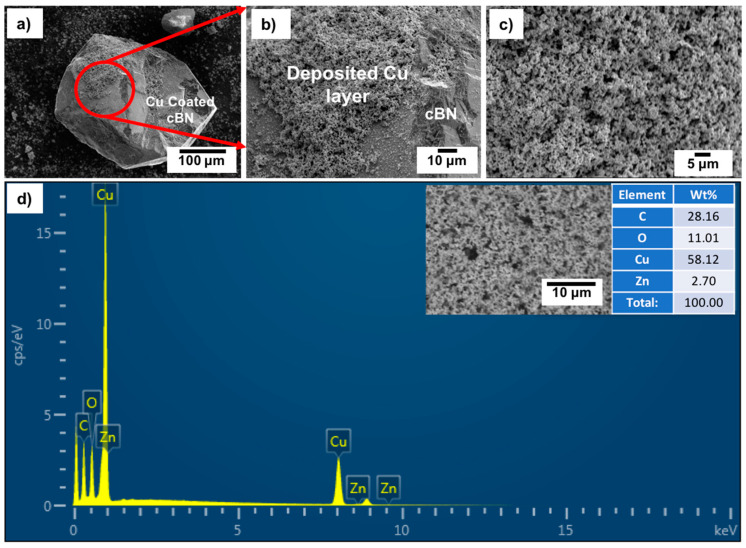
(**a**–**c**) FESEM images with different magnification and (**d**) EDAX compositional spot analysis of the un-metallized cBN/Cu composite particles (C peak is the background).

Figure 7a–c shows FESEM images with low and high magnifications of the surface of the un-metallized cBN/Cu composite particles. It was observed from the results that the cBN abrasive particles are completely encapsulated with a spongy copper layer composed of nanosized copper particles. Figure 7d shows an EDAX compositional analysis of the deposited copper layer. It was revealed that the deposited layer is composed mainly of elemental copper. However, there were no significant peaks of boron and nitrogen due to the homogeneous encapsulation of the cBN particles with the copper layer. The appearance of the oxygen peak indicates some oxidation of some nanosized copper particles by forming copper oxide, which can be identified by the X-ray diffraction, as explained in the next section.

**Figure 8:**
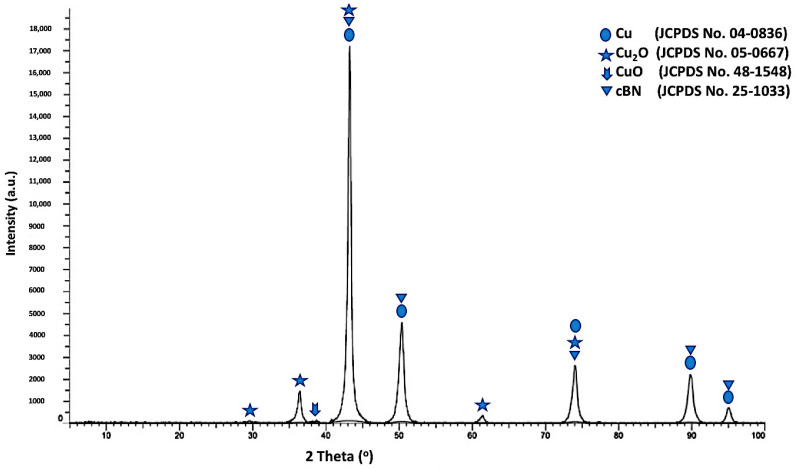
X-ray diffraction pattern of the un-metallized cBN/Cu composite particles.

Figure 8 shows the X-ray diffraction pattern of the un- metallized cBN/Cu composite particles. It was revealed from the results that four types of peaks were detected. The first type includes five peaks—(111), (200), (220), (311) and (222)—corresponding to the JCPDS card number 04-0836 for the (fcc) copper metal as a main crystalline phase, indicating the formation of the copper phase [19]. The second type of peak, which appeared due to the presence of the low oxidation state oxide of copper (Cu_2_O) crystalline phase, includes the five peaks corresponding to the JCPDS card number 05-0667 The third type of peak, which corresponds to the JCPDS card number 48-1548, is the low intense peak, which revealed the presence of the high oxidation state oxide of copper (CuO) crystalline phase. Finally, the fourth type includes the five peaks that revealed the presence of the 10 wt. % of the polycrystalline cubic boron nitride (cBN). It was also observed that the three peaks of the (200) at (2θ = 50°), (220) at (2θ = 74°) and (311) at (2θ = 90°) for the coated cBN by copper had lower diffraction intensities than the corresponding uncoated cBN (see Figure 4). This may be due to the effect of the layer of the deposited copper nanoparticles that covers the surface of the cBN and changes the orientation of the interacting X-ray beams with the surface of the cBN. Beside this reason, the intensity can be affected by the presence of the 10-weight percent of the cBN particles, which are coated with 90 wt.% of the copper nanoparticles. Moreover, the crystallite size of the deposited metallic copper nanoparticles on the surface of the cBN particles was calculated by the X-ray line broadening method at the highest intensity main peak of 2 *θ* = 43° using Scherrer’s formula (*D* = 0.9 λ/*B* cos *θ*), and the Scherrer crystallite size was estimated at 1.8 × 10^2^ Å [20,22].

**Figure 9:**
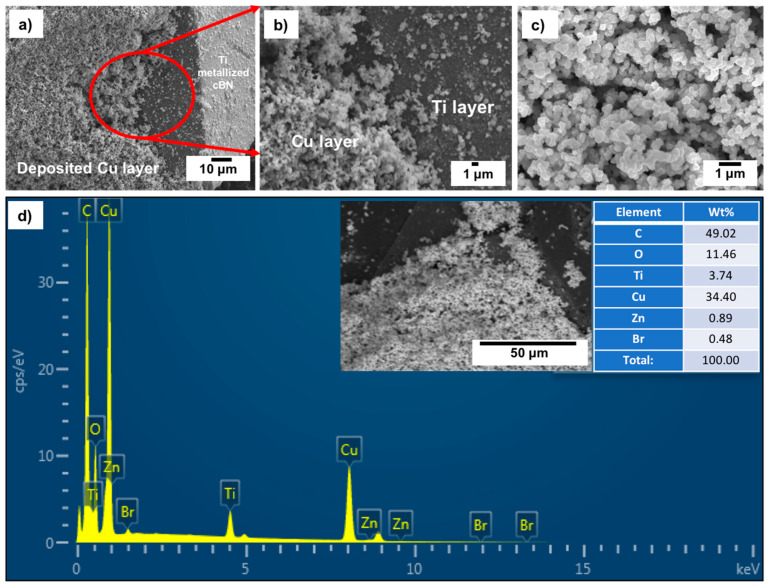
(**a**–**c**) FESEM images with different magnification and (**d**) EDAX compositional spot analysis of the Ti-metallized cBN/Cu composite particles (C peak is the background).

Figure 9a–c shows FESEM images with different magnifications of the Ti-metallized cBN/Cu composite particles. It was revealed from the results that the Ti-metallized cBN particles were coated with a condensed copper layer composed of a nanosized copper particles deposited on the nanosized Ti-layer, which metallized the cBN particles. Figure 9d shows an EDAX compositional analysis of the deposited copper layer on the Ti-metallized cBN particles. It was observed that the deposited layer on the surface of the Ti-metallized cBN abrasive particles was composed mainly of elemental copper; the presence of the Ti peaks was due to the presence of the Ti layer on the cBN particles, which revealed that the alkaline tartrate solution of the electroless copper bath does not effect the layer of the deposited Ti metal on the surface of the cBN particles. In addition, the appearance of the oxygen peak indicates the oxidation of some nanosized copper particles deposited on the surface of the cBN particles. Moreover, the disappearance of the boron and nitrogen peaks revealed that the deposited copper on the surface of the cBN was mainly composed of a thick layer of nanosized copper, which decreased the interaction between the electron beams and the encapsulated cBN particles by copper during the EDAX analysis.

**Figure 10:**
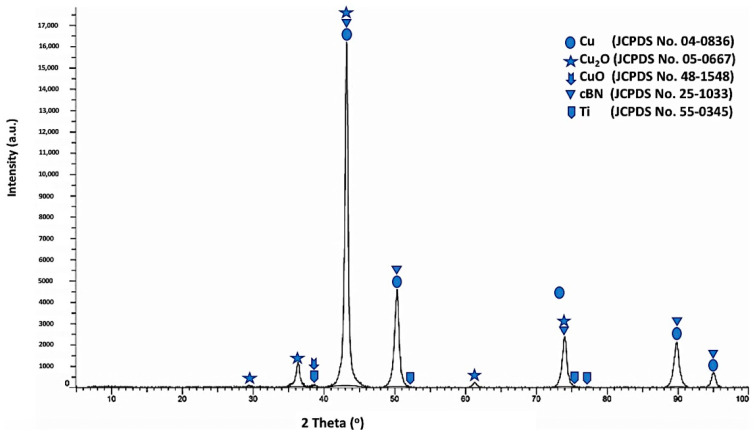
X-ray diffraction pattern of the Ti-metallized cBN/Cu composite particles.

Figure 10 shows the X-ray diffraction pattern of the Ti-metallized cBN/Cu composite particles. Five types of diffraction peaks were observed from the results. The first type included five peaks (111), (200), (220), (311) and (222) for the (fcc) copper metal as a main crystalline phase, which revealed the formation of the copper phase [20,21,22]. The second type of peaks is the five peaks that appeared due to the presence of the crystalline phase of the low oxidation state oxide of copper (Cu_2_O). The third type of peaks is the low intense peak that revealed the presence of the crystalline phase of the high oxidation state oxide of copper (CuO). The fourth type of peaks includes the five low intense peaks that revealed the presence of the metallic Ti phase corresponding to the JCPDS card number 55-0345. However, the fifth type includes five peaks that indicated the presence of the 10 wt.% of the polycrystalline cubic boron nitride (cBN) phase. It was also observed from the results that the three peaks of the (200) at (2θ = 50°), (220) at (2θ = 74°) and (311) at (2θ = 90°) for the copper coated Ti-metallized cBN had lower diffraction intensities than the corresponding ones of the un-metallized cBN (see Figure 4). This may be due to the effect of the Ti/Cu multilayer deposited on the surface of the cBN, which covered the surface of the cBN and changed the orientation of the incident X-ray beams with the surface of the cBN. Moreover, the crystallite size of the deposited copper on the surface of the cBN abrasive particles was calculated by the X-ray line broadening method using Scherrer’s formula (*D* = 0.9 λ/*B* cos *θ*) at the highest intensity main peak of 2 *θ* = 43°, and the Scherrer crystallite size was estimated 1.8 × 10^2^ Å [22].

**Figure 11:**
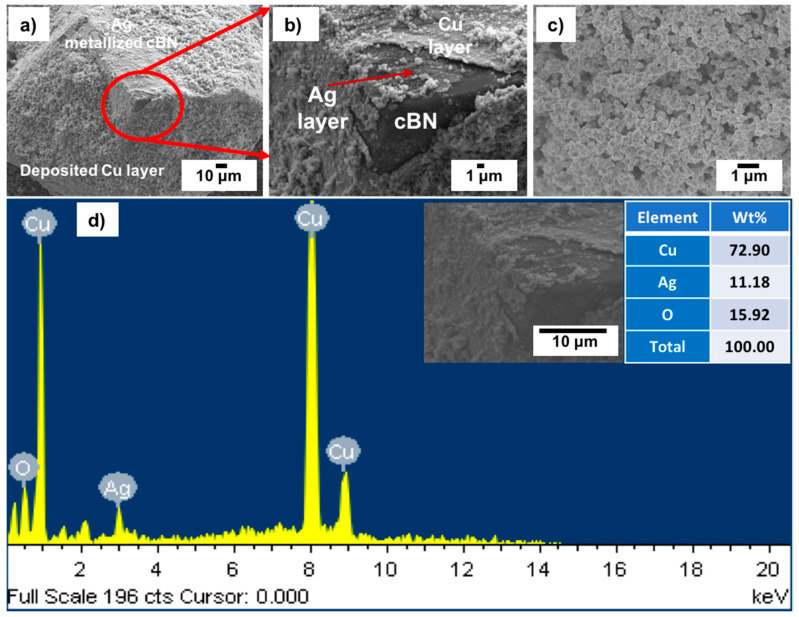
(**a**–**c**) FESEM images with different magnification and (**d**) EDAX spot compositional analysis of the Tin/Silver-metallized cBN/Cu composite particles.

Figure 11a–c shows FESEM images with low and high magnifications of the tin/silver-metallized cBN/Cu composite particles. It was observed from the results that the cBN particles were completely encapsulated within a uniform copper layer composed mainly of copper nanoparticles. Figure 11d shows an EDAX compositional analysis of the deposited copper layer on the tin/silver-metallized cBN abrasive particles. It was revealed that, the deposited layer on the surface of the tin/silver-metallized cBN abrasive particles was composed mainly of elemental copper. The presence of the silver peak confirmed the presence of the initial layer of the deposited Ag nano-layer on the surface of the cBN particles. It was expected that this Ag layer enhanced the autocatalytic reaction of the electroless deposition of Cu on the cBN particles. The alkaline tartrate solution of the electroless copper bath did not affect the deposited Ag layer on the surface of the cBN particles. The peaks of boron and nitrogen also disappeared, due to the effect of the deposited copper layer, which had enough thickness to cause the electron beams to reach the surface of the cBN particles during the EDAX analysis. Moreover, the presence of the oxygen peak indicated the formation of some oxides of the copper layer deposited on the surface of the cBN particles.

**Figure 12:**
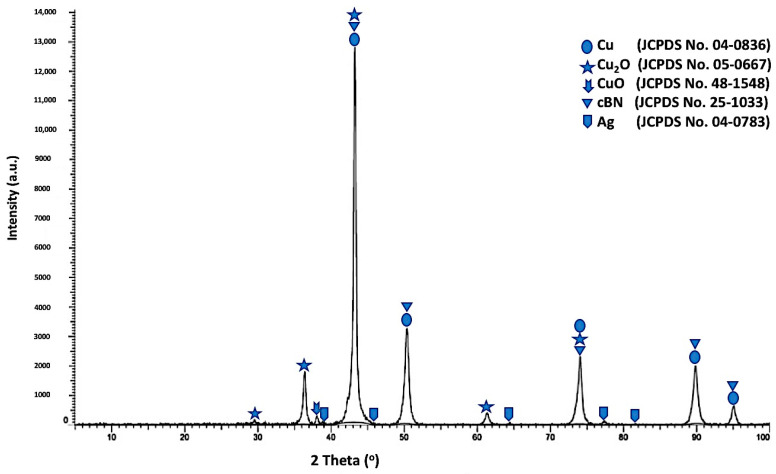
X-ray diffraction pattern of Tin/Silver-metallized cBN/Cu composite particles.

Figure 12 shows the X-ray diffraction pattern of the tin/silver-metallized cBN/Cu composite particles. Five types of diffraction peaks were detected. The first type includes five peaks, (111), (200), (220), (311) and (222) for the (fcc) copper metal as a main phase, which revealed the formation of the crystalline copper phase. The second type of peaks includes the five peaks that appeared due to the presence of the copper oxide (Cu_2_O) phase [16]. The third type of peak is the low intense peak that revealed the presence of copper oxide (CuO) phase. In addition, the fourth type includes five low intensive peaks that revealed the presence of (111), (200), (220), (311) and (222) of the silver nanolayer corresponding to the JCPDS card number 04-0783. They were initially deposited on the surface of the cBN particles during the tin/silver metallization process [19,20,22,23]. In addition, four peaks of the polycrystalline cubic boron nitride were observed. The results also showed that the three peaks of the (200) at (2θ = 50°), (220) at (2θ = 74°) and (311) at (2θ = 90°) for the coated cBN by copper have lower diffraction intensities than the corresponding uncoated cBN (see Figure 4). This is due to the effect of the deposited silver, as well as the deposited copper multilayers, which covered the surface of the cBN by thick layers and which affected the interaction of the X-ray beams with the surface of the cBN particles. The crystallite size of the deposited copper on the surface of the tin/silver-metallized cBN/Cu composite particles was calculated at the highest intensity main peak of 2 *θ* = 43° by the X-ray line broadening method using Scherrer’s formula (*D* = 0.9 λ/*B* cos *θ*), and the Scherrer crystallite size was estimated at 2.3 × 10^2^ Å. The crystallite size of the deposited copper nanoparticles on the tin/silver-metallized cBN/Cu composite particles increased due to the formation of the silver nanoparticle on the interface between the cBN and the deposited copper metal. It was also observed from the diffraction pattern of the tin/silver-metallized cBN/Cu composite particles that there was not any new or foreign phase detected by the XRD that could be formed during the coating process [21,22,23,24].

It was also observed from the study of the morphology of the deposited copper layers on the metallized cBN particles by the two different methods that the tin/silver-metallized cBN particles were mostly encapsulated by a condensed copper layer. However, the un-metallized cBN particles were encapsulated by a spongy and loose copper layer bonded with the surface of the un-metallized cBN particles. On the other hand, the Ti-metallized cBN particles were coated with an adhered copper layer.

#### 3.2.4. Thermo-Gravimetric Analysis (TGA) of the cBN/Cu Abrasive Particles

**Figure 13:**
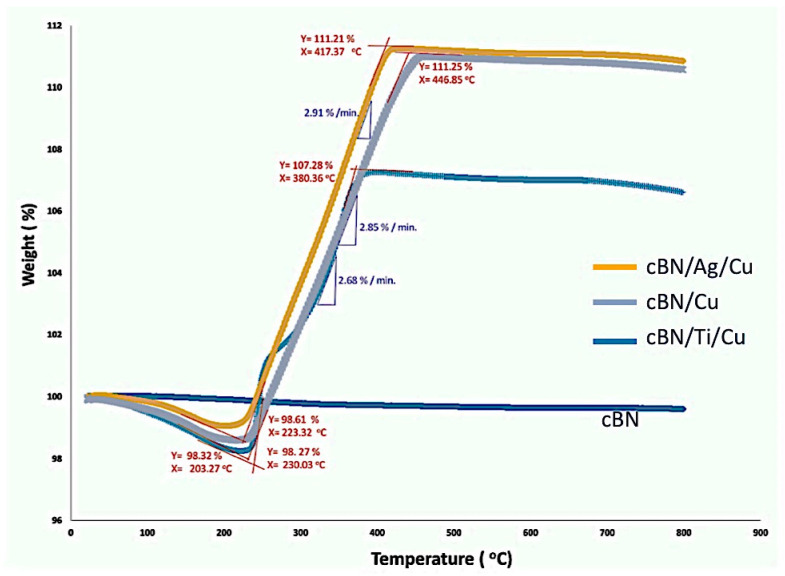
TGA curves of the uncoated cBN abrasive particles as well as the produced 10 wt.% cBN/Cu composite powders by different metallization techniques followed by the electroless deposition process.

The uncoated polycrystalline cBN particles, as well as the prepared 10 wt.% cBN/Cu composite abrasive powders, underwent thermogravimetric analysis to study the stability and durability of the cBN/Cu composite abrasive particles against high temperature. The TGA curves of the investigated powders under static nitrogen atmospheres as a purge gas are shown in Figure 13. A TGA thermogravimetric curve is displayed from left to right. The descending TGA thermal curve indicated that weight loss occurred. The extrapolated onset points (where the change of the weight begins) and the slope of each curve were estimated by the extrapolation method. The results revealed that the weight of the uncoated polycrystalline cBN abrasive particles remained unchanged at temperatures up to the investigated temperature of 800 °C. Only about 0.2% of the decrease in weight is mainly due to the loss of any adsorbed gases and water contents trapped in the pores, groves or cracks on the surface of the cBN particles, as shown in the SEM micrograph in Figure 3b.

On the other hand, the results of the TGA of the produced 10 wt.% cBN/copper composite particles with the different metallization methods of Ti, as well as tin/silver coated with the electroless copper deposition methods, revealed that the weight gain of the tin/silver-metallized cBN/copper and the un-metallized cBN/copper composite samples of ~12.6% and 12.98% started at 223 °C and 230 °C until the temperatures reached 417 °C and 446 °C, respectively, with a similar rate of ~2.9 wt%./min. On the other hand, the Ti-metallized cBN/copper composite sample weight gain of about 8.96% started at 203 °C until the temperature reached 380 °C by a lower rate of ~2.6 wt%./min. The reason behind the increment in the weight gain of all the produced samples may be due to the interaction between the deposited copper nanoparticles on the surface of the cBN particles and the atmospheric gases, such as oxygen, as well as nitrogen, during heating in the furnace of the TGA apparatus. However, the reason behind the difference in the weight gain % may be due to the high stability of the titanium metallic layer against the interactions with the atmospheric conditions more than the copper layer and the deposited silver layer at the same experimental conditions. By raising the heating temperature up to 800 °C, all the produced cBN/copper composite samples remained without any change in weight and only slightly decreased, with no great significant mass loss or gain, by raising the temperature above ~446 °C until reaching 800 °C. This slight change in the weight % may be due to the decomposition of any remaining copper oxides included on the surface of the copper coated cBN particles [19]. In addition, the intermetallic compounds composed of the CuN_3_, Cu_3_N and CuN_6_ may be formed due to the interaction between the nanosized copper particles and the cBN particles, as well as the nitrogen gas of the heating atmosphere at high temperature up to the investigated temperature of 800 °C [13,22].

## 4. Conclusions

In this investigation, 10 wt.% cBN/ copper composite abrasive particles were prepared by electroless copper deposition at room temperature. The nonconductive surface of the cBN particles was metallized by depositing a Ti or Ag nanosized conductive layer on the surface before coating with copper. The molten salt process was optimized to metallize the surface of the cBN particles by a dense and uniform nanosized layer of Ti. The deposited Ti layer was homogenously distributed on the surface of the cBN particles. It was observed that the copper electroless deposition reaction was enhanced. On the other hand, the tin/silver metallization method was used to activate and metallize the cBN abrasive particles by a nanosized Ag layer. The deposited silver nanoparticles promoted the autocatalytic reaction on the surface of the cBN particles and enhanced the electroless deposition process. The metallized, as well as the un-metallized, cBN particles underwent surface encapsulation using the electroless copper deposition method. cBN particles were successfully coated with a uniform layer of copper. The loading of copper coating layer on the cBN abrasive particles was optimized up to 90 wt.%. A thermogravimetric analysis of the investigated particles was conducted to study the stability of the particles at high temperature. The TGA data revealed that the Ti-metallized 10 wt.% cBN/copper composite particles had higher stability at temperatures up to 800 °C compared to the un-metallized and the tin/silver-metallized 10 wt.% cBN/copper composite particles, respectively. This suggests that the combination of both the metallization process by the Ti–molten salt reaction and the electroless copper deposition processes are very versatile; the Ti and Cu metallic layers that were deposited on the surface of the cBN particles increased their stability at high temperature more than the un-metallized and the tin/silver-metallized 10 wt.% cBN/copper composite particles.

## Data Availability

Not applicable.
